# Communication in Hong Kong Accident and Emergency Departments: The Clinicians’ Perspectives

**DOI:** 10.1177/2333393615576714

**Published:** 2015-03-31

**Authors:** Eloise Chandler, Diana Slade, Jack Pun, Graham Lock, Christian M. I. M. Matthiessen, Elaine Espindola, Carman Ng

**Affiliations:** 1 The International Research Centre for Communication in Healthcare, The Hong Kong Polytechnic University, Hong Kong SAR, China

**Keywords:** emergency care, communication, health care professionals, health care, interprofessional, relationships, health care, empathy, interviews, semi-structured

## Abstract

In this article, we report findings from the first qualitatively driven study of patient–clinician communication in Hong Kong Accident and Emergency Departments (AEDs). In light of the Hong Kong Hospital Authority’s policy emphasis on patient-centered care and communication in the public hospitals it oversees, we analyze clinicians’ perceptions of the role and relevance of patient-centered communication strategies in emergency care. Although aware of the importance of effective communication in emergency care, participants discussed how this was frequently jeopardized by chronic understaffing, patient loads, and time pressures. This was raised in relation to the absence of spoken interdisciplinary handovers, the tendency to downgrade interpersonal communication with patients, and the decline in staff attendance at communication training courses. Participants’ frequent descriptions of patient-centered communication as dispensable from, and time-burdensome in, AEDs highlight a discrepancy between the stated Hong Kong Hospital Authority policy of patient-centered care and the reality of contemporary Hong Kong emergency practice.

It is now internationally recognized that effective communication in health care is integral to ensuring patients’ safety and satisfaction with the quality of care they receive. Over the last two decades, researchers have successively linked effective patient–clinician communication with positive patient health outcomes, including patients’ satisfaction with, and confidence in, clinicians, adherence to recommended treatment, and accurate diagnosis (see, for example, Coleman et al., 2013; Crane, 1997). Ineffective communication, whether between patients and clinicians, or between clinicians themselves, still remains, however, a leading and preventable cause of patient harm, across health settings (Chiu & Chung, 2000; Wood, Sutton, Clark, McKeon, & Bain, 2006).

To address this, governing health bodies and hospitals around the world now promote models of patient-centered care and through it patient-centered communication as the most effective and safe model of health care delivery (see, for example, D. H. Lau, 2002; U.K. Department of Health, 2013). Patient-centered care has been correlated with greater levels of patient satisfaction with, and informed involvement and participation in, their treatment (Buckley et al., 2013; Nitzan et al., 2012; Rider et al., 2014; Slade et al., 2011). As Hobgood, Riviello, Jouriles, and Hamilton (2002) wrote, patient-centered care positions the “patient’s experience of the illness” on equal footing with the treatment of their health complaint or disease, and recognizes the development of rapport and empathy between patients and clinicians, through sustained interpersonal communication, as essential to securing effective patient–clinician alliances (p. 1258).

In Hong Kong, the Hospital Authority (the governing body for the administration and management of Hong Kong public hospitals) has also sought to advance a model of patient-centered care and communication. It posits patient-centered care as a core value driving health care delivery in the hospitals it oversees, and cites effective “two-way communication” as “indispensible for understanding and meeting a patient’s needs” (Hong Kong Hospital Authority, 2013). Moreover, in its *Strategic Plan 2012–2017*, the Authority has prioritized improving and promoting patient-centered communication among hospital clinicians (Hong Kong Hospital Authority, 2012). Despite this policy emphasis, miscommunication and/or communication breakdowns between clinicians and patients and between clinicians themselves continue to be identified as leading causes of critical incidents in Hong Kong hospitals (see, for example, Hong Kong Hospital Authority, 2014) and patients’ dissatisfaction (Griffiths & Yeoh, 2011). Hong Kong Accident and Emergency Departments (AEDs) have, in particular, been singled out as warranting greater clinician communication training. Recently, in the first population-based Hong Kong Patient Satisfaction Survey, more than 20% of AED patient respondents cited receiving inadequate or no information about their diagnosis and treatment prior to being discharged (Griffiths & Yeoh, 2011, p. 109).

From a research perspective, AEDs provide unique contexts in which to study clinician–patient communication. Patients typically present as strangers to the AED, with no readily accessible medical records or established relationships with the clinicians (nurses or doctors) who will be treating them (Chung, 2005; Hobgood et al., 2002; Slade et al., 2011). Perhaps more than any other site within the health care system, emergency care relies heavily on effective spoken communication between patients and clinicians as the former articulate their symptoms and concerns and the latter draw on this to complement physical examination, diagnosis, and subsequently negotiate treatment (Redfern, Brown, & Vincent, 2009). However, the importance of communication, and in particular interpersonal communication between clinicians, patients, and patients’ families, in emergency care is often downplayed (see, for example, Gordon, Sheppard, & Anaf, 2010, p. 82).

To date, Hong Kong–based researchers have investigated the effectiveness of communication within AEDs through a quantitative lens, assessing the quality of clinicians’ communication practices through retrospective analyses of patients’ complaints or surveys of patients’ satisfaction (F. L. Lau, 2000; Tam & Lau, 2000). We sought to address this gap in qualitative research in the Hong Kong emergency care context by undertaking the first qualitatively driven investigation of communication practices between clinicians and patients, and among clinicians, in a Hong Kong AED. Adopting the methodology and methods of Slade et al.’s (2011) ethnographic and linguistic research on AED communication within Australia, the aims of our research were to describe and analyze spoken communication between clinicians and patients, identifying the ways in which clinicians could enhance their communicative practices to improve the quality and safety of the patient experience. We combined qualitative and quantitative ethnographic methods (Creswell, 1998; Gumperz & Hymes, 1972; Silverman, 2001) to uncover the socio-cultural context, with linguistically oriented discourse analysis of the audiotaped interactions between patients and clinicians in the AED (based on the model of Eggins & Slade, 2005). This involved five components: (a) observations, (b) semi-structured interviews with clinicians, (c) a questionnaire administered to clinicians, (d) audio-recordings of patients’ journeys from triage to disposition, and (e) brief follow-up interviews with patients.

In this article, we report on the second and third components of this study, presenting the views of clinician participants—what they identified as important in their communication with patients and colleagues, their reported strategies for communicating effectively, their views on the relevance of interpersonally sensitive and patient-centered communication in their work, and what they perceived as impeding efficient and effective communication with patients in the AED (see Slade, Chandler, et al., 2015, for an analysis of audio-recorded clinician–patient communication). Our findings present a complex picture of how Hong Kong clinicians perceive the role of communication within AEDs, the relevance to them of patient-centered communication within emergency care, and the tensions between the Hospital Authority’s stated policy of patient-centered care and what they as emergency clinicians believe is important and possible in practice.

## Methods

This study was conducted in one of the most highly attended AEDs in Hong Kong. The AED had a daily attendance rate of more than 600 patients, providing emergency care to approximately 231,000 per year (Hong Kong Hospital Authority, 2012). It employed 43 doctors and 62 nurses at the time of data collection. Ethics approval for conducting this research was obtained through the Hong Kong Polytechnic University Human Research Ethics committee. Twenty-eight AED clinicians participated in semi-structured interviews, and 58 completed questionnaires. Both the interviews and questionnaires elicited participants’ views on communication in the AED.

### The Questionnaire

We developed the questionnaire following a review of international and Hong Kong health communication research. The design was also influenced by previous research by Slade et al. (2011) within the Australian AED context. In the questionnaire, we first asked a series of closed demographic questions concerning respondents’ professional roles, age, and years working within the AED. We then posed 15 open-ended and closed questions, crafted to elicit respondents’ views and experiences of communication problems within the AED. Questionnaire data were statistically analyzed using SPSS.

Twenty-two doctors and 36 nurses completed the questionnaire. Respondents from both professions had varying degrees of seniority and work experience within the AED: 21% had worked in the AED for 2 years or less (*n* = 12), 27% for between 3 and 6 years (*n* = 16), and 52% for 7 years or more (*n* = 30). All identified Cantonese as their first language.

### The Interviews

After conducting a preliminary analysis of questionnaire data, we identified emergent trends and themes across the response set, and used these to inform the drafting of the interview questions. The interview was semi-structured, designed to provide interviewees with opportunities to elaborate on their experiences and opinions of communication issues in the AED. Broadly, interviewees were asked: to reflect on the role of communication in their work; what barriers or challenges, if any, they perceived to effective communication with patients; what communication strategies they adopted when interacting with patients; and what they perceived to be impediments to implementing patient-centered care within the AED context. Drawing on the bilingual proficiency of members of the research team, interviewees were given the option of participating in English or Cantonese. Interviewees were also invited to choose whether they participated jointly with a colleague or individually.

With interviewees’ consent, the interviews were audio-recorded, transcribed, and de-identified. Transcripts of interviews conducted in Cantonese were subsequently translated into English by two members of the research team. A third researcher performed a final check for accuracy of translation and transcription against the original audio-recordings. In the analysis of the transcripts, a grounded-theory approach was taken (Glaser, 1992). In other words, we tried to approach the data with as few preconceived theoretical notions as possible and to allow themes to emerge from the analysis (Bowen, 2006). From these themes we constructed our analytical framework. Such thematic analysis “involves the search for and identification of common threads that extend throughout an entire interview or set of interviews” (Morse & Field, 1995, p. 139). To do this, we made use of NVivo, software designed for in-depth qualitative analysis. We first read through the transcripts carefully and gave an initial coding to all segments relevant to any aspect of communication in the AED. We then carried out several rounds of comparing, sorting, and recoding as we looked for connections among coded segments and compared analyses developed from one part of the interview data both with other parts of the interview data and with the questionnaire data. In this way, a number of major themes emerged relating to the clinicians’ views of the nature and quality of communication in the AED.

Ultimately eight doctors and 20 nurses participated in interviews, including senior departmental managers, specialists, ward managers, and junior clinicians. Six interviews were conducted jointly (with 12 interviewees), and 16 individually (with 16 interviewees). Interviewees’ work experience in emergency care ranged from 1 year to 23 years in the case of nurses and from 2 years to more than 25 years in the case of doctors. Fifteen interviews were conducted in Cantonese. The remainder were in English. All interviewees had previously completed the questionnaire.

## Findings

Below we present findings from our analysis of questionnaire and interview data. We have arranged our discussion of these findings into three broad thematic categories: *organizational, informational*, and *interpersonal* (see Eggins & Slade, 2012; Halliday & Matthiessen, 2013; Matthiessen, 2013a, 2013b; Slade & Matthiessen, in press). Organizational themes concern how participants’ perceive institutional factors such as staff shortages, workloads, patient loads, and AED policies impact their ability to communicate effectively with patients. Informational themes relate to participants’ accounts of the means and modes through which patients’ medical information is transferred among clinicians, through clinical handover, and between clinicians and patients in the context of the Hong Kong multilingual AED. And, interpersonal themes concern clinicians’ views on the importance of interpersonally sensitive, patient-centered communication in their interactions with patients. Organizational factors, such as patient quotas, institutional policies, and staffing do inevitably influence and affect both the manner of information transfer and interpersonal communication. However, where a theme primarily related to challenges associated with the communication of patient information, we categorized it as informational, and where it primarily concerned an issue of how clinician–patient relationships were built and maintained though communication, we categorized it as interpersonal. These thematic categories have been influenced by two of Halliday’s semantic metafunctions—experiential and interpersonal (see Halliday & Matthiessen, 2013). Halliday’s interpersonal function refers to the language choices that enable speakers to enact interpersonal relationships. The experiential function refers to the language choices that speakers use to construe their experience of the world. Broadly, we are capturing the distinction between the two domains of “how” we talk to develop interpersonal relationships and the informational content of “what” we talk about. Organizational themes are the institutional, contextual factors that constrain these two domains of talk.

### The Organizational Themes: The Perceived Impact of Patient Loads, Understaffing, and Time Pressure on Communication

Researchers who have previously studied communication in AEDs have cited increased patient demand and (in parallel) staff shortages as posing serious communication obstacles between patients and clinicians—obstacles which, if not overcome, can jeopardize patients’ safety (see, for example, Rhodes et al., 2004). In our study, this was reinforced, with participants most frequently citing time pressure, understaffing, and patient loads as the key challenges they faced in effectively communicating with patients and other clinicians throughout their work.

Many interviewees (in particular nurse interviewees) discussed the impact of long working hours on staff morale and how this, when coupled with increased patient loads and demand, often resulted in very tense and regimented interactions with patients in their care. All interviewees, both doctors and nurses, regardless of discipline, level of seniority, or length of experience in the AED, at some point reflected on how time limitations and the nature of the emergency service they were working within tested their communication skills. This concern is supported by the questionnaire findings. More than 50% of questionnaire respondents cited long working hours as either “always” or “sometimes” negatively impacting on their ability to communicate effectively (*n* = 31).

As one nurse interviewee remarked, the AED had “the highest attendance [rate] in Hong Kong, but . . . the lowest nurse manpower” (Senior Nurse, Interview). Several interviewees reported working at minimum 12-hour days throughout their working week. Others discussed being regularly called into work on pre-organized leave days. This level of demand on staff was confirmed by nearly 90% of questionnaire respondents. A reported roll on effect of the level of understaffing at the AED reported in the interviews was that clinicians were no longer being encouraged or permitted to undertake communication training, unless they did so during their personal time. Indeed, two managerial staff who participated in a joint interview stated that the high degree of understaffing within the AED made it virtually impossible for them to release their staff to attend the communication training courses available and promoted by the Hospital Authority: “I cannot assign the basic manpower to the clinical [work] . . . How can I take out some nurse for training?” (Senior Nurse, Interview). These managers made it clear that while the staff shortage continued, clinicians’ attendance to communication training courses would continue to decline (again, the onus would be on clinicians to undertake such training independently): “We have so many courses for communication tailor-made for nurses, doctors or other staff . . . but we don’t have time to send the nurses to go to those” (Senior Nurse, Interview). Notably, they did so while acknowledging the links that research has made between clinicians’ attendance and patient satisfaction, and indeed the Hospital Authority’s current policy emphasis on improving clinicians’ patient-centered communication skills.

### Informational Themes: Reflections on the Spoken and Written Transfer of Patients’ Medical Information among Clinicians and Between Clinicians and Patients Across Languages

Interviewees’ reflections on the degree to which organizational factors affected their abilities to effectively communicate with patients were very closely linked to further discussions on the means and modes through which patients’ information was communicated among clinicians and in patient–clinician interactions. This was particularly apparent in interviewee discussions of clinical handover practices, that is, how, what, and when patient information and responsibility was transferred between clinicians over the course of a patient’s journey through the AED.

### Challenges to Effective Clinical Handover Between Disciplines and Departments

Clinical handover in this AED was described as single disciplinary (i.e., nurse to nurse, doctor to doctor), and often delivered through written notes on medical files as opposed to face-to-face communication. That said, interviewees reported no prescribed or regular method of handing over patient information to each other: verbal or written, informal or formal—although face-to-face handover was stated by one doctor as the preferred method for medical staff if time allowed. Handover between nurses was described by many interviewees as a relatively ad hoc or even “rare” practice dictated by the time pressures and patient loads of each shift.

Formal handover between nurses and doctors was described as exceptional by interviewees of both disciplines. Rather, interdisciplinary communication in the course of the AED patient’s journey was reported as occurring through brief written notes on the patient’s files. This was often justified by reference to what Redfern et al. (2009) describe as the “unbounded nature” of the AED: the 24-hour, non-stop presentation of patients and the effect this has on clinicians’ time to communicate with each other (p. 653). In this way, interdisciplinary handover was portrayed as impractical or even a luxury of other health care realms—which in the AED context interrupted direct patient care:

. . . if you want to have a handover, you may spend at least 15 to 20 minutes; but patients still keep coming in! You can’t stop your service for the handover . . . so it will be practically very difficult for us to do a formal handover between doctors and nurses. (Senior Doctor, Interview)

Among questionnaire respondents, only 26% (*n* = 15) reported problems associated with interdisciplinary communication. The vast majority, (72%, *n* = 42) did not perceive the current practice as problematic. Nonetheless, the routine absence of interdisciplinary handover or structured interdisciplinary communication arguably did affect the quality of the care patients received. Nurse interviewees regularly reported that they would often find patients who had not been provided with appropriate information on the treatment and medication they would receive and in some cases were not informed of their discharge instructions, or indeed whether they were being discharged from the AED at all. With doctors assuming that nurses would take on the “cover-up” role and nurses assuming that doctors would take charge of explanation (without communicating this to one another), patients would not receive information on post-consultation treatments and the subsequent steps involved in their care trajectories. Thus, while interdisciplinary handovers were often dismissed as timewasting, their absence created further time-burdens, with nurses often describing having to “chase” doctors to obtain the unwritten explanations of diagnosis and treatment. Omissions in information-giving were reported as particularly prevalent at discharge which, according to our follow-up patient interviews, sometimes resulted in patients’ lack of understanding of their diagnosis and thereby lack of compliance with treatment. From one nurse’s perspective, this was accentuated in situations of patients’ admissions to hospital wards, at which time “it’s not likely that a lot of [patients] get to see the doctor” (Interview).

Nurses were not alone in reporting inadequate information-giving to patients. As one doctor commented,

because we have to deal with too many cases . . . time does not allow us to explain a very clear diagnosis . . . We usually explain very preliminary results and then actually there’s a follow-up plan, but we do not have time to express or discuss [it]. (Interview)

Some nurses accepted that part of their role was to take on greater patient education responsibilities as a consequence of the greater organizational burdens placed on emergency physicians: “The doctors . . . have a quota. They need . . . to see the patient and then they have to see the next one. So maybe they don’t have time to educate the patient more” (Registered Nurse, Interview); others perceived this as falling beyond the scope of their knowledge and responsibilities: “Actually if you ask me, I think . . . it’s the doctor’s responsibility [to explain] why we need to do such treatment . . . Because s/he is the person to order treatment, they should be the ones to explain” (Registered Nurse, Interview). For several, regardless of the doctor’s intentions to move on to other patients, in situations where they felt ill equipped to provide patients with adequate explanations they would ultimately insist on the doctor re-visiting the patient.

Although these interviewees did not expressly link these interruptions in continuity of care to failures in structured interdisciplinary handover or communication, many did remark on communication difficulties and potential safety issues occurring in the handing over of patient information and responsibility between departments. The AED was variously described as a “factory,” a “manufacturing line,” a demanding workspace that had developed its own unique “common language,” sometimes inhibiting inter-departmental information exchange. One interviewee provided a recent anecdote in which the AED staff did not handover a patient’s medication to staff in the operation theatre, where the medication was to be administered. Although this error was ultimately noticed and the patient received the medication, the patient’s surgery was unnecessarily delayed. A questionnaire respondent reported a similar incident. Information on drugs previously taken by a patient was overlooked during handover between departments. Without this information, medical staff administered an additional dose of the same medicine. One third of questionnaire respondents (*n* = 19) reported adverse incidents “sometimes” occurring because of failures in communication during handover. This, when coupled with interviewees’ accounts of handover practices within the AED in general (most of which downplayed the significance of interdisciplinary handover to patient safety and continuity of care while also anecdotally suggesting avoidable errors or omissions in patient care did indeed result) suggests that further research is warranted exploring the extent to which contemporary Hong Kong handover practices might negatively impact on patients’ AED treatment (see Eggins & Slade, 2012).

### Communicating Across Languages: The Hong Kong Multilingual AED

Another theme which emerged relating to the transfer of information across the AED was the challenge of communicating patient information and treatment across languages in the multilingual Hong Kong context. Hong Kong AED clinicians often communicate bilingually, sometimes trilingually, throughout their work in Cantonese, Mandarin, and English. This reflects the official languages of Hong Kong and the tertiary education of clinicians, who, if trained locally, would have done so predominantly in English. Consultations with patients tend to be in Cantonese or Mandarin, mirroring the native language of the majority of patients. Spoken communication between clinicians over the course of patients’ care will generally take place in Cantonese with English code switching and mixing when medical terminology is used. English is however the primary language used in written documentation—patient files and medical charts. As noted above, interdisciplinary communication between doctors and nurses generally occurs through this written medium. If one traces the flow of patients’ information over the course of their care trajectories, it is transferred not only through two modes (written and spoken) but also back and forth between a multidisciplinary team of clinicians and the patient, and across languages. As one senior doctor described it, the multilingual nature of Hong Kong AED work can be seen in “two parts. One is you get the history from [the] patient in Cantonese and then you need to write in English. The second part is we learn in English, we tell the patient in Cantonese . . . ”

Slightly more questionnaire respondents reported information sometimes becoming lost or changed in the translation of English medical knowledge to spoken Cantonese when communicating with patients (48%, *n* = 28) than when translating patient provided information in Cantonese to written English medical records (45%, *n* = 26). Interviewees were similarly more likely to describe difficulty in communicating English medical diagnoses in Cantonese to patients. This was largely put down to there often being no corresponding Cantonese phrases to the English medical terms. One way clinicians discussed overcoming this was to consult an English–Cantonese glossary. Where this failed, clinicians described adopting descriptive and “everyday” Cantonese terms to provide their patients with more accurate understandings of their conditions or treatments.

When clinicians encountered difficulties in the translation of spoken Cantonese to written English in patients’ files, they described a degree of flexibility in the written conventions. It had become accepted practice to incorporate the Cantonese terms within the otherwise English medical files. Greater difficulty was reported as occurring when the electronic records system was used, with one interviewee suggesting a need for the technology to be upgraded to allow for the recognition of Chinese medical terminology.

For the majority of doctors and nurses we interviewed, however, the greatest language challenge they faced was when communicating with patients from migrant populations who had limited Cantonese or English language skills. Although interpreting services are available to the hospital at large, they are not immediately available to the AED team, who by the nature of their work must assess patient presentations as quickly and efficiently as possible. This was seen as a particular problem in triage, with one nurse commenting that it at times posed safety issues for patients who were unable to communicate the nature of their illness: “If they’re in emergency situation, they need urgent care and urgent treatment; but we delay it [because] of the language barrier” (Senior Nurse, Interview).

Many interviewees reported that when communicating with non-native Cantonese or English speakers, they would greatly simplify their language and, where this failed, rely on physical cues to ascertain the nature of the presenting patient’s ailment (see, for example, Kang & Zayts, 2013, on the importance of gesture in effective health communication between clinicians and patients with language barriers). For some doctors, the presence of a translator, while necessary, created its own challenge to effective communication and the establishment of rapport and trust between clinicians and patients. The doctors’ and patients’ reliance on interacting with each other through a third party was seen as interrupting the one-on-one relationship between doctor and patient, and posing an additional risk to information loss through the translator’s intervention.

Below we address the interpersonal themes which emerged in our analyses. This dimension concerns how clinicians variously discussed the AED patient–clinician relationship and the relevance, or importance, of patient-centered care and patient-centered communication in emergency care.

### The Interpersonal Themes: Perceptions of the Patient–Clinician Relationship, the Importance of Empathy and Rapport, and Managing Patient Expectations

Interviewees provided divergent characterizations of the relationship between AED staff and patients. Some described their relationship with patients in business terms: “We are delivering a service to our patients . . . They’re actually our clients” (Senior Doctor). Rather than minimizing the importance of interpersonal communication with patients under their care, those who perceived AED patients as consumers of a medical service were often more likely to view securing patient satisfaction with the health care they received through building rapport as an essential component of their work. A significant number of interviewees, however, did frame building empathy and rapport with patients as a privilege, one that was more available to clinical staff outside the AED. Often this was expressed by reference to the shorter timeframes within which patients received care within the department. (In contrast, 45% (*n* = 26) of questionnaire respondents rated the importance of establishing empathy and rapport with patients as “high,” 53% (*n* = 31) described it as of “medium” importance. Only 22% (*n* = 13) cited the impact of time pressures on communication as unique to emergency medicine.)

Following a patient’s discharge, whether home or to another hospital ward, their relationship with AED clinicians will often cease. This distinctly finite relationship between clinicians and patients meant that developing empathy and rapport was de-prioritized by some interviewees.

Since . . . we don’t get to see the patients again after treatment, there’s no rapport, really. We’re just with each other for a few minutes. Even for assessments, it’s just going to take half a day. So relatively speaking . . . if the patients trust you, they’ll trust you [more] next time; but it isn’t long-time [patient] care . . . compared with other specialties, [empathy] isn’t very important. (Resident Doctor, Interview)

Indeed, some nurses reflected on how they would purposively minimize interpersonal communication with patients in an effort to get through the triage waiting list as quickly as possible.

Nurses will not introduce themselves or their role in triage, because triage is so busy . . . they don’t want to talk too much to the patient, because if you build up rapport with the patient, they will ask, they will talk more and more. We have no time to . . . do that. (Senior Nurse, Interview)

Triage is the first stage in patient care within the AED. It represents the first encounter patients will have with clinicians and as such sets the stage for patients’ impressions of the quality of health service they will receive (Cameron et al., 2010, p. 617). Somewhat ironically then, the functional nature of the triage process often means that of all stages throughout the patient journey, it is here that interpersonal communication strategies are most likely to be sacrificed. The goal of triage is to ascertain as quickly as possible the urgency of the patient’s ailment and then categorize patient cases accordingly. Communication thus tends to take the form of working through a checklist—clinician led and confined, task oriented, and fast paced (Slade et al., 2011; Slade, Manidis, et al., in press). Notably, when asked whether effective communication could be challenging in triage, 64% (*n* = 37) of questionnaire respondents stated “sometimes” and 19% (*n* = 11) stated “always.” As one nurse interviewee described it,

[W]hen we . . . work in triage, we handle one case in two or three minutes. . . . Therefore we have . . . very little time to talk with the patient, . . . educate our patient or even to reassure our patient . . . We need to . . . concentrate on our assessment, history taking, and also the vital sign taking. Other than that, we are in a hurry for the next turn. (Senior Nurse, Interview)

At least two nurse interviewees expressly rejected patient and policy expectations that they adopt more patient-centered communication strategies in triage, with one indicating that to do so would subject them to extensive criticism from their nursing peers:

You tell me, you need time to build up empathy. If I spend 15 minutes to build empathy with the patient, then later the nursing officer will . . . complain [to] me . . . “you don’t do triage. Instead of doing the talking or the counseling the patient—it’s not your role—you have to make the triage first.”

For several of those working beyond the triage desk, however, the time-strapped nature of emergency care led to the reverse view: developing rapport and empathy was essential to ensure not only patient satisfaction but also patient safety in terms of accurate diagnoses and patient compliance with treatment. For these interviewees, seeking out the patient’s agenda was regarded as the most important step in achieving rapport with, if not in delivering the best care to, their patients. Many interviewees however discussed a conflict between patients’ expectations and what was possible to achieve within the AED context. For some nurses, the unpredictable nature of the AED terrain made it difficult to always provide patients with the information they sought.

We aren’t familiar with the patients . . . We have a reference frame [but] sometimes you just can’t answer what they ask. Sometimes . . . they ask things that are in the far future: “ . . . Can s/he recover? Can s/he walk again?” These, [questions we] can’t really give an answer [to] . . . (Registered Nurse, Interview)

For several doctors, episodes of patient dissatisfaction with the AED staff stemmed from mismatches between patients’ expectations of the kind of treatment offered and the reality of emergency medicine.

A lot of patients think that they’re not dealing with emergency cases in the AED . . . Once they arrive, they say that they have to screen this and that, take a look at this and that. Because they can’t afford the private [clinics], they’re here . . . So I think how it turns out is that they come here [and] we cannot offer them [what they expect] . . . Sometimes they may have waited for a few hours and turns out they don’t get much in the end. (Resident, Interview)

Managing patients’ expectations of the nature of AED treatment was thus seen by many as integral to ensuring more effective and positive staff–patient interactions. While the Hong Kong Hospital Authority has introduced measures directed at improving patients’ understanding of the precise role and nature of AED services (take for example, the online patient service guides which explain the triage system and advise against AED attendance in circumstances of non-emergency care: see Hong Kong Hospital Authority, n.d.), based on interviewee discussions, patient orientation documents (posters, leaflets) readily available and disseminated within the AED were frequently not read.

A few triage nurses we interviewed did discuss the small (and time-cognizant) measures they adopted to help patients understand the AED process and let them know their presence was not being ignored by staff, despite lengthy waiting times. These interviewees reported that they would often perceive a marked difference in patients’ attitudes and expressed frustrations with the AED system and staff following verbal acknowledgment, however brief, of the patient’s discomfort. Indeed, as one nurse observed, the expression of empathy often had a more positive impact on patient satisfaction than just attending to their physical needs:

[Establishing empathy and rapport is] important [so that] patients feel that you care about him/her or not . . . [If we] go to him/her and say, “Mm, I understand you’re not well. How about we get you a bed for rest?” it’s better than you take a bed to him/her. I’m still going to get a bed after saying that; but s/he will be happier than if I just grabbed a bed and blankets. (Senior Nurse, Interview)

Another recommended patient-centered care strategy for orienting patients to AEDs and putting them at ease is for staff to introduce themselves to patients—letting them know who they are and what their role is (Slade et al., 2011). AED patients will often encounter not only staff of different disciplines but also staff from different departments. For the uninitiated patient, it will not always be apparent whether their attending clinician is a nurse or a doctor, particularly as AED nurses will often perform minor operations, traditionally associated with the doctor role. Only a minority of interviewees reported adopting this strategy as one they utilized to build rapport with their patients.

Providing adequate explanations is crucial in establishing empathy and rapport, one reason being that the more informed patients are, the more in control they may feel of what is often an unsettling and alien situation. Central to this is listening to patients, opening up the space to allow them to describe their concerns, which might be missed in more clinician-dominated consultations (Slade et al., 2011). This step also forms the basis of engaging patients and encouraging their participation in consultations. Interviewees in the present study, however, varied in their approach to promoting patient participation in AED treatment decisions. For some, patient participation was expected:

I . . . firmly believe that . . . patient management is something that patients themselves should participate [in] . . . If they want to make their decisions, they have the intellectual [ability] to participate in the decision-making, I’d definitely involve them, give them information to let them make their own decisions. (Senior Doctor, Interview)

As the above quote indicates, however, the extent to which this was encouraged by doctors was often based on their individual assessments of patients’ desires to do so and their health literacy:

I think for different people, what you tell them may be different . . . if s/he is quite smart, or s/he has a higher education level . . . then perhaps what you talk about would be different. And you have to know how much the patient knows about his/her condition. Some things are very hard to understand; I wouldn’t complicate things too much. I mean, simplify things as much as possible, to let him/her understand better. (Junior Doctor, Interview)

Notably, clinicians who reported routinely engaging patients in treatment decisions did not do so necessarily because of a belief in the patient’s right to be truly informed in their health care or pointedly because of any policy agenda in favor of patient participation. Rather, these clinicians adopted patient-participation strategies because they perceived positive health outcomes associated with this practice. As one senior doctor observed, by providing patients with comprehensive information on the reasons for treatment and subsequent discharge instructions, patients would be less likely to return to the AED, “because if they don’t have enough information, they will come back very soon . . . for the same illness” (Senior Doctor, Interview). These clinicians’ observations align with findings of previous studies which have linked poor information-giving practices and lack of patient consultation in treatment decisions with prolonged stays in, or unscheduled returns to, emergency care (see, for example, Clancy, 2009).

## Discussion

The unique AED context inevitably poses challenges to effective communication both between clinicians and patients and among clinicians themselves. As the above findings demonstrate, the combined stress of understaffing, patient demand, and resulting time constraints were omnipresent in all interviewees’ reflections on their communication within AEDs, whether this related to perceptions of the relationships between patients and clinicians, the communication strategies adopted by clinicians in the course of their work, and even interdisciplinary handover.

Interpersonally sensitive, effective communication is, however, an essential component of safe and efficient AED clinical practice, which not only results in greater levels of patient satisfaction, but has also been demonstrated as key to ensuring optimal clinical outcomes including more efficient and accurate diagnosis (Slade et al., 2011) and patient adherence to treatment following discharge (see, for example, Nitzan et al., 2012, p. 115). Senior management reports that conditions of understaffing, particularly among nurses, and patient demand have reached the point where they are now unable to find the time or resources to encourage or even release their staff to attend communication training are of significant concern.

While patient-centered care is promoted by the Hospital Authority as the model of care adopted within the hospitals it oversees, the degree to which it was perceived as important in the AED context or was being implemented by participating clinicians varied. As discussed above, questionnaire respondents and interviewees provided mixed views on the degree they believed building empathy and rapport with their patients was relevant to, or possible, in their work. While some reported routinely incorporating interpersonal strategies in their patient interactions, such as introducing themselves, discovering and responding to patients’ agendas and expectations, taking the time to explain AED processes and treatment, and encouraging patient participation in their treatment decisions, all qualified this by reference to what patient loads and time allowed (see also Lam & Webster, 2009). However, as Hobgood et al. (2002) observed, successive international studies on clinician–patient communication within AEDs have shown that doctors often “underestimate the amount of information desired by the patient while overestimating the time spent explaining and planning in the interview by up to 900%” (p. 1298).

Encouraging patient participation in decision-making has been linked to greater patient co-operation with treatment plans and subsequent declines in rates of patient returns. Locally, several researchers have investigated the receptiveness of the Hong Kong population to the idea of patient participation in health care decision-making. In Bennett, Smith, and Irwin’s (1999) study of Hong Kong residents’ preferences with respect to models of health care consultations, the vast majority of participants favored shared decision-making between doctors and patients (p. 269). In Kim, Smith, and Yueguo’s (1999) comparative study of Beijing and Hong Kong residents’ desires for joint decision-making, the Hong Kong cohort overwhelmingly preferred models of patient participation. Similarly in Henderson and Chien’s (2004, 2007) exploration of Hong Kong surgical patients’ expectations of participation in treatment decision-making, all participants preferred input in decisions on surgical intervention. However, among interviewees in the present study, only a few doctors and nurses reported routinely engaging their patients in the course of their treatment. Those who did reported modifying the degree to which they would do so based on their own assessments of patients’ desires and health literacy.

Research conducted elsewhere has demonstrated that while clinicians may modify the amount of information they provide patients based on such assessments, this often does not align with patients’ own desires for information, regardless of their background (Cramm & Dowd, 2008, p. 365). Indeed, in the fourth and fifth components of our research—the audio-recordings of patients’ journeys from triage to disposition, and the follow-up interviews with patients—patients repeatedly expressed their desire for greater information about their AED treatment to relieve their anxiety and to feel that they were being treated and recognized as participants in their health care. The recordings of clinician–patient interactions also demonstrated that for several patients, the interpersonal dimensions of their care were frequently neglected. In these cases, all patients later discussed their frustrations with not having been listened to, or comforted, by clinicians, with one patient stating to researchers on her discharge from the AED that as a result she would not be complying with the AED’s discharge instructions.

## Conclusion

Despite demonstrating an awareness of the Hospital Authority’s patient-centered communication policy agenda and to a more limited extent, the importance of effective communication in AED care, all staff interviewees reflected on how conditions of increased patient overload, chronic understaffing, and resulting time pressures frequently jeopardized their communication with patients and with each other. This was particularly raised in relation to the virtual abandonment of spoken handover practices between nursing and medical staff, dismissed as impractical given time constraints, the downgrading of interpersonal communication and information-giving practices (integral to patient-centered care) with patients, and the reduction in staff attendance to communication training courses because of staff shortages. Communication training, however, is essential to ensure AED staff develop the skills to competently and safely transfer patient information between each other, as well as to ensure that patients have a comprehensive understanding of their diagnosis and post-discharge treatment instructions. The common positioning of patient-centered communication as dispensable, time-burdensome, and distinct from emergency care by participants (and in particular interviewees) in this study highlights a discrepancy between stated Hong Kong Hospital Authority policy and the reality of contemporary Hong Kong emergency practice. Greater resources need to be allocated to communication training to ensure AED clinicians’ abilities to incorporate interpersonally sensitive, patient-centered communication in their interactions with patients, and improve their understanding of how this is integral to the quality, safety, and efficiency of emergency care.

## References

[bibr1-2333393615576714] Bennett K. Smith D. H. Irwin H. (1999). Preferences for participation in medical decisions in China. Health Communication, 11, 261–284. doi:10.1207/S1532702 7HC110308

[bibr2-2333393615576714] Bowen G. A. (2006). Grounded theory and sensitizing concepts. International Journal of Qualitative Methods, 5, Article 2. Retrieved from ualberta.ca/~iiqm/backissues/5_3/pdf/bowen.pdf

[bibr3-2333393615576714] Buckley B. A. McCarthy D. M. Forth V. E. Tanabe P. Schmidt M. J. Adams J. G. Engel K. G. (2013). Patient input into the development and enhancement of ED discharge instructions: A focus group study. Journal of Emergency Nursing, 39, 553–561. doi:10.1016/j.jen.2011.12.018 22575702

[bibr4-2333393615576714] Cameron K. A. Engel K. G. McCarthy D. M. Buckley B. A. Mercer Kollar L. M. Donlan S. M.. . . Adams J. G. (2010). Examining emergency department communication through a staff-based participatory research method: Identifying barriers and solutions to meaningful change. Annals of Emergency Medicine, 56, 614–622. doi:10.1016/j.annemergmed.2010.03.017 20382446

[bibr5-2333393615576714] Chiu H. S. Chung C. H. (2000). Risk management in emergency department. Hong Kong Journal of Emergency Medicine, 7, 96–103.

[bibr6-2333393615576714] Chung C. (2005). Meeting the challenges of rising patient expectations: The 10 “C”s for emergency physicians. Hong Kong Journal of Emergency Medicine, 12, 3–5.

[bibr7-2333393615576714] Clancy C. M. (2009). Reengineering hospital discharge: A protocol to improve patient safety, reduce costs, and boost patient satisfaction. American Journal of Medical Quality, 24, 344–346. doi:10.1177/1062860609338131 19502567

[bibr8-2333393615576714] Coleman E. A. Chugh A. Williams M. V. Grigsby J. Glasheen J. J. McKenzie M. Min S.-J. (2013). Understanding and execution of discharge instructions. American Journal of Medical Quality, 28, 383–391. doi:10.1177/1062860612472931 23354870

[bibr9-2333393615576714] Cramm K. J. Dowd M. D. (2008). What are you waiting for? A study of resident physician-parent communication in a pediatric emergency department. Annals of Emergency Medicine, 51, 361–366. doi:10.1016/j.annemergmed.2007.09.032 18006190

[bibr10-2333393615576714] Crane J. A. (1997). Patient comprehension of doctor-patient communication on discharge from the emergency department. The Journal of Emergency Medicine, 15, 1–7. 9017479 10.1016/s0736-4679(96)00261-2

[bibr11-2333393615576714] Creswell J. W. (1998). Qualitative inquiry and research design: Choosing among five traditions. Newbury Park, CA: SAGE.

[bibr12-2333393615576714] Eggins S. Slade D. (2005). Analysing casual conversation. London: Equinox.

[bibr13-2333393615576714] Eggins S. Slade D. (2012). Clinical handover as an interactive event: Informational and interactional communication strategies in effective shift-change handovers. Communication & Medicine, 9, 215–227. doi:10.1558/cam.v9i3.215 24575676

[bibr14-2333393615576714] Glaser B. G. (1992). Basics of grounded theory analysis: Emergence vs forcing. Mill Valley, CA: Sociology Press.

[bibr15-2333393615576714] Gordon J. Sheppard L. A. Anaf S. (2010). The patient experience in the emergency department: A systematic synthesis of qualitative research. International Emergency Nursing, 18, 80–88. doi:10.1016/j.ienj.2009.05.004 20382369

[bibr16-2333393615576714] Griffiths S. Yeoh E. (2011). Patient satisfaction survey 2010. Retrieved from http://www.ha.org.hk/ho/pred/pssreportforweb.pdf

[bibr17-2333393615576714] Gumperz J. J. Hymes D. H. (1972). Directions in sociolinguistics: The ethnography of communication. New York: Holt, Rinehart and Winston.

[bibr18-2333393615576714] Halliday M. A. K. Matthiessen C. M. I. M. (2013). Halliday’s introduction to functional grammar (4th ed.). Abingdon, UK: Routledge.

[bibr19-2333393615576714] Henderson A. Chien W.-T. (2004). Information needs of Hong Kong Chinese patients undergoing surgery. Journal of Clinical Nursing, 13, 960–966. doi:10.1111/j.1365-2702.2004.01004.x 15533102

[bibr20-2333393615576714] Henderson A. Chien W.-T. (2007). Health beliefs and expectations implicit in decision-making in a Hong Kong Chinese surgical population. Journal of Clinical Nursing, 16, 603–609. doi:10.1111/j.1365-2702.2006.01572.x 17335536

[bibr21-2333393615576714] Hobgood C. D. Riviello R. J. Jouriles N. Hamilton G. (2002). Assessment of communication and interpersonal skills competencies. Academic Emergency Medicine, 9, 1257–1269. 12414480 10.1111/j.1553-2712.2002.tb01586.x

[bibr22-2333393615576714] Hong Kong Hospital Authority. (2012). Annual report 2011-2012. Retrieved http://www.ha.org.hk/upload/publication_13/435.pdf

[bibr23-2333393615576714] Hong Kong Hospital Authority. (2013). Vision, missions & values. Available from http://www.ha.org.hk

[bibr24-2333393615576714] Hong Kong Hospital Authority. (2014). Annual report on sentinel and serious untoward events: 1 October 2012–30 September 2013. Retrieved from http://www.ha.org.hk/haho/ho/psrm/ECOPYSEREPORT2014.PDF

[bibr25-2333393615576714] Hong Kong Hospital Authority. (n.d.). Guide to accident & emergency service. Available from http://www.ha.org.hk

[bibr26-2333393615576714] Kang M. A. Zayts O. A. (2013). Interactional difficulties as a resource for patient participation in prenatal screening consultations in Hong Kong. Patient Education & Counseling, 92, 38–44. doi:10.1016/j.pec.2013.01.008 23414659

[bibr27-2333393615576714] Kim M. S. Smith D. H. Yueguo G. (1999). Medical decision making and Chinese patients’ self-construals. Health Communication, 11, 249–260. doi:10.1207/S15327027 HC110307

[bibr28-2333393615576714] Lam M. Webster J. (2009). The lexicogrammatical reflection of interpersonal relationship in conversation. Discourse Studies, 11, 37–57. doi:10.1177/1461445608098497

[bibr29-2333393615576714] Lau D. H. (2002). Patient empowerment: A patient-centered approach to improve care. Hong Kong Medical Journal, 8, 372–374. 12376717

[bibr30-2333393615576714] Lau F. L. (2000). Can communication skills workshops for emergency department doctors improve patient satisfaction? Journal of Accident & Emergency Medicine, 17, 251–253. 10921810 10.1136/emj.17.4.251 PMC1725405

[bibr31-2333393615576714] Matthiessen C. M. I. M. (2013a). Appliable discourse analysis. In Fang Y. Webster J. J. (Eds.), Developing systemic functional linguistics: Theory and application (pp. 135–205). London: Equinox.

[bibr32-2333393615576714] Matthiessen C. M. I. M. (2013b). Applying systemic functional linguistics in healthcare contexts. Text & Talk, 33, 437–466.

[bibr33-2333393615576714] Morse J. M. Field P. A. (1995). Qualitative research methods for health professionals. Thousand Oaks, CA: SAGE.

[bibr34-2333393615576714] Nitzan U. Hirsch E. Walter G. Lurie I. Aviram S. Bloch Y. (2012). Comprehension and companionship in the emergency department as predictors of treatment adherence. Australasian Psychiatry, 20, 112–116. doi:10.1177/ 1039856211432485 22461657 10.1177/1039856211432485

[bibr35-2333393615576714] Redfern E. Brown R. Vincent C. A. (2009). Identifying vulnerabilities in communication in the emergency department. Emergency Medicine Journal, 26, 653–657. doi:10.1136/emj.2008.065318 19700582

[bibr36-2333393615576714] Rhodes K. V. Vieth T. He T. Miller A. Howes D. S. Bailey O.. . . Levinson W. (2004). Resuscitating the physician-patient relationship: Emergency department communication in an academic medical center. Annals of Emergency Medicine, 44, 262–267. doi:10.1016/S0196064404001994 15332069

[bibr37-2333393615576714] Rider E. A. Kurtz S. Slade D. Esterbrook Longmaid H. E. III Ho M-J. Pun J.. . . Branch W. T. (2014). The International Charter for Human Values in Healthcare: An interprofessional global collaboration to enhance values and communication in healthcare. Patient Education & Counselling, 96, 273–280. doi:10.1016/j.pec.2014.06.017 25103181

[bibr38-2333393615576714] Silverman D. (2001). Interpreting qualitative data: Methods for analyzing talk, text, and interaction. Newbury Park, CA: SAGE.

[bibr39-2333393615576714] Slade D. Chandler E. Pun J. Lam M. Matthiessen C. M. I. M. Williams G.. . . Tang K. S. (2015). Effective healthcare worker-patient communication in Hong Kong Accident & Emergency Departments. Hong Kong Journal of Emergency Medicine.

[bibr40-2333393615576714] Slade D. Manidis M. McGregor J. Scheeres H. Stein-Parbury J. Dunston R.. . . Herke M. (2011). Communicating in hospital emergency departments: Final report. Sydney: University of Technology, Sydney.

[bibr41-2333393615576714] Slade D. Manidis M. McGregor J. Scheeres H. Chandler E. Stein-Parbury J.. . . Herke M. (in press). Communication in hospital emergency departments. New York: Springer.

[bibr42-2333393615576714] Slade D. Matthiessen C. M. I. M. (in press). The texture of casual conversation: A multidimensional interpretation (Two volumes). London: Equinox.

[bibr43-2333393615576714] Tam A. Y. B. Lau F. L. (2000). A three-year review of complaints in emergency department. Hong Kong Journal of Emergency Medicine, 2, 16–21.

[bibr44-2333393615576714] UK Department of Health. (2013). Treating patients and service users with respect, dignity and compassion. Retrieved from https://www.gov.uk/government/policies/treating-patients-and-service-users-with-respect-dignity-and-compassion

[bibr45-2333393615576714] Wood R. Sutton M. Clark D. McKeon A. Bain M. (2006). Measuring inequalities in health: The case for healthy life expectancy. Journal of Epidemiology & Community Health, 60, 1089–1092. doi:10.1136/jech.2005.044941 17108308 PMC2465513

